# Quality of life in individuals with neurofibromatosis type 1 associated cutaneous neurofibromas: validation of the Dutch cNF-Skindex

**DOI:** 10.1186/s41687-024-00732-w

**Published:** 2024-05-29

**Authors:** Britt A. E. Dhaenens, Sarah A. van Dijk, Laura Fertitta, Walter Taal, Pierre Wolkenstein, Rianne Oostenbrink

**Affiliations:** 1 Department of General Paediatrics, Erasmus MC-Sophia Children’s Hospital, Wytemaweg 80, Rotterdam, 3015 CN The Netherlands; 2 The ENCORE Expertise Centre for Neurodevelopmental Disorders, Erasmus MC, Rotterdam, The Netherlands; 3 Department of Neurology, Erasmus MC Cancer Institute, Dr. Molewaterplein 40, Rotterdam, 3000 CA The Netherlands; 4 Department of Dermatology, National Referral Center for Neurofibromatosis, Henri Mondor University Hospital, Assistance Publique– Hôpitaux de Paris, Creteil, France; 5 Full Member of the European Reference Network on Genetic Tumour Risk Syndromes (ERN GENTURIS), Nijmegen, The Netherlands

**Keywords:** Neurofibromatosis type 1, Cutaneous neurofibroma, Quality of life, QoL, Patient-reported outcome measure, cNF-Skindex

## Abstract

**Background:**

Almost all patients with Neurofibromatosis type 1 (NF1) develop cutaneous neurofibroma (cNF), benign dermal tumours that have a large impact on the patient’s Quality of Life (QoL). The French cNF-Skindex is the first questionnaire to specifically assess cNF-related QoL in patients with NF1. We aimed to adapt and validate a Dutch version of the cNF-Skindex.

**Methods:**

The questionnaire was translated using forward and backwards translation, and subsequently administered to a sample of 59 patients on two separate occasions. Feasibility was evaluated by the presence of floor/ceiling effects. Reliability was assessed by evaluating internal consistency and test-retest reliability, by calculating Cronbach’s alpha and Spearman’s rank correlation coefficients. The EQ-5D-5L and SF-36 were used to evaluate convergent validity, using Spearman’s rank correlation coefficients. An exploratory factor analysis was performed to study the data’s internal structure. Multivariable linear regression was used to model the relationship between patient characteristics and the cNF-Skindex.

**Results:**

The Dutch cNF-Skindex demonstrated excellent feasibility and reliability (Cronbach’s alpha 0.96, test-retest correlation coefficient 0.88). Convergent validity was confirmed for the EQ-5D-5L and relevant SF-36 scales. All items and subdomains from the original questionnaire were confirmed following exploratory factor analysis. The patient characteristics included in the multivariable linear regression were not significantly associated with the cNF-Skindex score.

**Conclusions:**

The Dutch cNF-Skindex displayed excellent psychometric properties, enabling use in the Netherlands.

**Supplementary Information:**

The online version contains supplementary material available at 10.1186/s41687-024-00732-w.

## Background

Neurofibromatosis type 1 (NF1) is a tumour predisposition syndrome with an estimated prevalence of 1 in 2.000 to 1 in 4.500 [1–3]. NF1 is associated with the development of nerve sheath tumours, including cutaneous neurofibroma (cNF). These benign tumours occur in nearly all individuals with NF1 and typically start developing during puberty. Their number tends to increase with age, ranging from a few to several thousand lesions [4]. cNF can cause symptoms like pain, itching, and disfigurement, and patients have reported that the visibility of the cNF negatively influences their body image [5]. Due to all these associated symptoms, cNF can negatively impact Quality of Life (QoL) [6, 7]. Recent studies showed that cNF located in the facial area and a higher number of cNF are associated with a lower cNF-related QoL [7, 8].

It has been increasingly common to include patient-reported outcomes (PRO) that assess QoL in clinical trials for cNF. While objective trial endpoints like the number and size of tumours provide information on physiologic disease severity, PROs reflect the patient’s perspective [9]. This is especially important in a chronic and variable condition like NF1, because the experienced morbidity due to cNF varies significantly between patients and within individual patients over time. The use of PROs in trials for NF1 enables patients to report their personal view on the impact of their condition. PROs can also provide invaluable information on the effect of investigational treatments in clinical trials and could guide decision making in clinical practice, as patients can express the effects of treatment on constructs such as QoL or specific symptoms like itching. Earlier studies have assessed cNF-related QoL in patients with NF1 using generic PROs like the SF-36 [10]. However, generic PROs often do not capture disease-specific problems and may be unable to detect subtle but relevant differences in QoL [11]. Evidence from the literature also indicates that disease-specific PROs have better psychometric properties than generic QoL measures [12–14]. Yet, the number of disease-specific PROs developed for NF1 has been limited.

In 2022, Fertitta et al. developed the first PRO specifically for cNF-related QoL in patients with NF1: the cNF-Skindex, an 18-item questionnaire that was modified from the original Skindex [8]. Being the first PRO designed to measure cNF-related QoL in individuals with NF1, the cNF-Skindex will be an invaluable outcome measure in both clinical trials and clinical practice. However, its usefulness in international settings is limited by the lack of adequate translations and cultural adaptation.

In the present study we aimed to translate the cNF-Skindex into Dutch, and to subsequently evaluate the psychometric properties of this translated version to enable use in the Netherlands.

## Methods

### Translation

Permission for translation was provided by the original author of the Skindex, Dr. M. Chren. An expert committee was formed, consisting of the principal investigator (PI) of this study, two forwards translators, and one backwards translator. The original French version of the cNF-Skindex was forward translated into the Dutch language by two translators, who were not aware of the content of the original questionnaire. Both translators had French as their native language and were fluent in Dutch. The study PI and forward translators subsequently compared the two translations, evaluating the translation from a clinical perspective and discussing any discrepancies until an agreement was reached. Next, the backward translation was performed by an individual bilingual translator with the Dutch nationality, who was fluent in French. The study PI compared the translation with the original version of the cNF-Skindex and discussed with the translators if any adaptations needed to be made to the Dutch version to retain consistency. This process resulted in the final version of the Dutch cNF-Skindex.

It was decided to not perform cognitive debriefing of the Dutch version of the cNF-Skindex, since the Dutch cNF-Skindex was nearly identical to the Dutch version of the Skindex-16 [15], an already validated instrument in Dutch. To create the cNF-Skindex, only minor changes were made to the original Skindex-16. For each item, the words ‘your skin’ were replaced with ‘your cutaneous neurofibroma’. In addition, two items were added that are relevant to cNF: (1) the influence of cNF on how patients experience touch, warmth, and cold on their skin, and (2) if the cNF get stuck on the patient’s clothing or hairbrush. In addition, patients would not have been allowed to participate in both the cognitive debriefing and psychometric evaluation stage of the study. Given the small pool of possible participants, it would not have been feasible to recruit adequate patient numbers for both stages. Hence, the psychometric evaluation stage was prioritised over cognitive debriefing.

### Psychometric evaluation

This study was performed at the Erasmus Medical Centre in Rotterdam, the Netherlands. The study was approved by the local Institutional Review Board (IRB) of the Erasmus Medical Center, local identifier MEC-2021-0598. All participants provided written consent to participate.

#### Recruitment of participants

Patients who met the revised diagnostic criteria for NF1 [16], aged 18 years or up, who had at least one cNF were recruited when they visited the outpatient Neurology clinic of the Erasmus Medical Centre from July 2022 through September 2023. Patients with severe comorbidities, defined as advanced malignant tumours during the time of the study, were excluded. When interested patients had not completed the questionnaires two weeks after the clinic visit, a reminder email was sent. A final telephone call was made as reminder four weeks after the clinic visit. Every responder was included in the analysis.

A digital version of the cNF-Skindex was administered to the participants using the data capture system Castor EDC. At the first administration, participants also completed a general information questionnaire, the EQ-5D-5L questionnaire [17] and the SF-36 questionnaire [18]. To study the test-retest reliability, participants were asked to complete the cNF-Skindex for a second time, 14 days after they completed the first administration.

#### Questionnaires used in this study

A general information questionnaire was used to collect sociodemographic variables of the participants, such as gender, age, marital status, education level, presence of learning problems, employment status, the estimated number of cNF (categories 0–10, 11–50, 51–100, 100+, based on Fertitta et al. [8]), general health perceptions, and the presence of other health problems.

The cNF-Skindex is an 18-item questionnaire, including three domains: “functioning”, “emotions”, and “symptoms” [8]. Each item is scored on a 7-point Likert scale (0 being never bothered, 6 being always bothered), with a recall period of seven days. The total score of the questionnaire ranges from 0 (no impairment of cNF-related QoL) to 108 (maximal impairment). A higher cNF-Skindex score indicates a poorer cNF-related QoL. The cNF-Skindex has three severity strata: patients with a total score from 0 to 11 can be considered as having non bothersome cNF, patients with a total score of 12 to 48 having moderately bothersome cNF, and patients with a total score of 49 or higher having importantly or very importantly bothersome cNF [19]. The cNF-Skindex has been validated in a French and US population of patients and showed good to excellent psychometric properties [8, 19].

The EQ-5D-5L is a brief and generic health status measure that comprises five dimensions: mobility, self-care, usual activities, pain/discomfort and anxiety/depression [17]. Each dimension has five Likert response options, ranging from ‘no problems’ to ‘extreme problems’. It has a one-day recall period. The answers given by the patient on the five dimensions are combined into a 5-digit number that describes the patient’s health state. The EQ-5D-5L also has a visual analog scale (the EQ-VAS) on which the patients can rate their own health from 0 to 100 (worst to best perceived health).

The 36-item Short Form Survey (SF-36) is a generic health-related QoL measure [18]. It comprises eight domains of health: physical function, limitations because of physical problems, limitations because of emotional problems, social function, mental health, energy, pain, and health perception. The item scores are recoded into percentages, ranging from 0 to 100, with higher scores indicating a more favourable health state. Items in the same domain are averaged together to create the eight scale scores. The version with the standard (4 weeks) recall period was used in this study.

#### Clinical information from electronic health records

Additional clinical information was extracted from the electronic health records of the participants, including the presence of NF1 diagnostic criteria, the presence of other NF1-related disease manifestations (osseous lesions, optic pathway glioma (OPG), other brain glioma, malignant peripheral nerve sheath tumour, pheochromocytoma), and the occurrence of any cNF-related interventions (surgical resection, CO2 laser, photocoagulation, electrodessication or diathermy).

#### Statistical analysis

Statistical analyses were performed using SPSS version 28.0. Non-parametric tests were used given the non-normal distribution of the data. The total and subdomain scores of the cNF-Skindex, EQ-5D-5L, and SF-36 were computed according to scoring instructions. The population was described using the mean, standard deviation (SD), and range for continuous variables, and count and frequency for categorical variables.

The feasibility of the Dutch version of the cNF-Skindex was analysed by assessing the presence of significant floor and/or ceiling effects for the total score and domain scores. Floor and ceiling effects were considered significant if ≥ 15% of the participants had the lowest or highest score. The internal consistency was evaluated by calculating Cronbach’s α, with a Cronbach’s α of ≥ 0.70 being considered adequate [20]. The test-retest reliability was determined by calculating the Spearman’s rank correlation coefficients between the scores of the first and second administration. A value of ≥ 0.75 indicates adequate test-retest reliability [21].

The Mann-Whitney U test was used to assess known-group validity between participants based on the number of cNF (≤ 50 or > 50), and the occurrence of cNF-related interventions. Convergent validity was assessed by calculating the Spearman rank correlation coefficients between the total and subdomain cNF-Skindex scores and the individual item scores of the EQ-5D-5L, the visual analogue scale of the EQ-5D-5L, and the SF-36.

An exploratory factor analysis was performed to study if the three domains of the French cNF-Skindex (emotions, symptoms, and functions) were confirmed in the Dutch version. Spearman’s correlation coefficients (r) for all items were computed. The factor analysis was conducted using promax rotation. The Kaiser–Meyer–Olkin (KMO) test was used to verify the sampling adequacy for the analysis. The scree plot and eigenvalues were used to justify the selection of the number of components. Component loadings of ≥ 0.4 were considered relevant [22].

Multivariable linear regression was performed to explore the association between the total cNF-Skindex score and the following factors: >50 cNF, age, sex, educational level (ISCED level), and the occurrence of any cNF-related intervention (yes/no). Educational level was included to control for the effect of cognitive impairment, which is present in 40–80% of the patients with NF1 [23–26], to correct for its influence on how patients interpret and complete the cNF-Skindex questionnaire.

The cNF-Skindex scores resulting from the present study were compared with the scores from the French population in which the cNF-Skindex was originally validated using independent t-tests [8].

## Results

Fifty-nine patients participated in the psychometric evaluation of the Dutch cNF-Skindex, and 50 participants completed the second administration of the cNF-Skindex (85%). The mean age of the participants was 46 years, and the majority was female (68%) (Table 1). The majority of participants reported having more than 100 cNF (48%), although the sample also included participants with 1–10 cNF (7%) and participants with 11–50 or 51–100 cNF (20% and 25%, respectively). Thirty-five participants (59%) had received one or more cNF-related intervention, mostly consisting of either surgical removal or treatment with CO2-laser.

**Table 1:** Socio-demographic information and clinical characteristics of the participant sample (*n* = 59)

		Mean (SD) or range	*n*	% of patients
**Socio-demographic information**				
Gender (male)			19	32%
Age (years)				
Mean (SD)		46 (14.4)		
Range		20–76		
Social status				
Married/living together			33	56%
Divorced/single			26	44%
ISCED education level				
Level 1–2			15	25%
Level 3–4			31	53%
Level 5–7			13	22%
**Clinical characteristics—general**				
Age at NF1 diagnosis (years)				
Mean (SD)		13 (12.1)		
Range		0–46		
NF1 diagnostic genetically confirmed			29	49%
De novo NF1 mutation			28	48%
Self-reported general health perception				
Bad or moderate			27	46%
Good or very good			32	54%
**Clinical characteristics—cutaneous neurofibroma**				
Number of cutaneous neurofibroma				
1–10			4	7%
11–50			12	20%
51–100			15	25%
100+			28	48%
Received therapy for cutaneous neurofibroma			35	59%
Surgical resection			24	41%
CO2 laser			18	31%
Other*			8	14%
**NF1 diagnostic criteria**				
Café-au-lait maculae			48	81%
Inguinal or axillary freckling			51	86%
Cutaneous neurofibroma			59	100%
Plexiform neurofibroma			24	41%
Optic pathway glioma			6	10%
Typical osseous lesion			1	2%
**Other NF1-related manifestations**				
Low grade brain glioma			4	7%
Scoliosis			16	27%
Self-reported presence of learning difficulties			32	54%

Typical osseous lesions are defined as sphenoid dysplasia, anterolateral bowing of the tibia, or pseudarthrosis of a long bone

*Other defined as photocoagulation, electrodessication or diathermy

The mean total cNF-Skindex score was 40.7 (Table 2). Nine participants had a total cNF-Skindex score that placed them in severity stratum 1 (nonbothersome cNF, 15%), 28 were in stratum 2 (moderately bothersome cNF, 48%) and 22 patients were in stratum 3 (very bothersome cNF, 37%). There were no significant ceiling effects, but one significant floor effect was observed for the ‘Functioning’ subdomain. The Cronbach’s alpha of the total score and the subdomains were all above 0.90, indicating high internal consistency.

Shapiro-Wilk tests confirmed that the total cNF-Skindex score and subdomain scores were not normally distributed (*p*-values ranging from < 0.001 to 0.003).

Adequate test-retest reliability was observed for both the total cNF-Skindex score (Spearman’s correlation *r* = 0.88), the ‘Functioning’ subdomain (*r* = 0.89), ‘Emotions’ subdomain (*r* = 0.83), and ‘Symptoms’ subdomain (*r =* 0.85), with all *p*-values < 0.001.

**Table 2:** Descriptive statistics and the Cronbach’s alpha for the total cNF-Skindex score and the three subdomains of the questionnaire

	Mean (SD)	Median (IQR)	Score range	Scoring minimum (%)	Scoring maximum (%)	Cronbach’s α
**cNF-Skindex**						
Total score	40.7 (27.1)	36.0 (15.0–65.0)	3–94	0	0	0.96
Functioning subdomain	10.0 (9.9)	6.0 (1.0–17.0)	0–32	**15**	0	0.93
Emotions subdomain	19.5 (11.0)	18.0 (9.0–31.0)	0–36	2	2	0.93
Symptoms subdomain	11.2 (9.1)	9.0 (3.0–20.0)	0–31	10	0	0.91

Values in bold = significant floor or ceiling effect

For known-group validity, significant differences in cNF-Skindex scores were observed between participants with ≤ 50 cNF or more than 50 cNF, as well as for participants who had and who had not received a cNF-related intervention (Table 3).

Regarding convergent validity, the mean Spearman correlation between the total cNF-Skindex score and the items of the EQ-5D-5L was 0.30 (correlations ranging between 0.07 and 0.358, with *p*-values ranging between 0.005 and 0.606) The Spearman correlation between the total cNF-Skindex score and the EQ-5D-5L VAS was − 0.32 (*p*-value = 0.012). The total and subdomain scores of the cNF-Skindex score also correlated significantly with several of the SF-36 scales (ANNEX 1).

The exploratory factor analysis revealed that the three domains as described in the original French study were confirmed. The Kaiser–Meyer–Olkin test verified the sampling adequacy for the analysis, with a KMO of 0.89. The scree plot showed an inflexion that justified the selection of three factors. The three identified factors (‘Functioning’, ‘Symptoms’, and ‘Emotions’) explained 76.4% of the total variance (ANNEX 2).

**Table 3:** Known-group validity of the Dutch cNF-Skindex

cNF-Skindex	≤50 cNF (n = 16)		>50 cNF (n = 43)		p-value	No cNF-related intervention (n = 24)		cNF-related intervention (n = 35)		p-value
	Mean	SD	Mean	SD		Mean	SD	Mean	SD	
Total score	27.9	24.4	45.4	26.8	**0.017**	30.5	20.9	47.7	28.9	**0.026**
Functioning scale	5.7	8.3	11.6	10.1	**0.032**	6.8	6.7	12.2	11.2	0.099
Emotions scale	13.7	11.2	21.7	10.2	**0.012**	14.9	9.0	22.7	11.2	**0.009**
Symptoms scale	8.6	7.9	12.2	9.4	0.175	8.8	8.1	12.9	9.6	0.098

The mean and standard deviation (SD) of the total and subdomain scores are given for each group, followed by the *p*-value as calculated by the Mann-Whitney U test. p-values < 0.05 in bold

Although univariate linear regression showed that having more than 50 cNF was associated with the total cNF-Skindex score (Table 4), when controlling for other characteristics such as age, sex, ISCED level and occurrence of a previous cNF-related intervention, this association was no longer statistically significant. This same result was seen for all three severity strata, as well as for each cNF-Skindex subdomain.

The overall mean cNF-Skindex score of the Dutch participant sample (40.7) was compared to the mean total score of the original French study population (47.2). T-test revealed that the mean total scores did not differ significantly between the Dutch and French population (*p*-value 0.070).

**Table 4:** Linear regression of the total cNF-Skindex score, presenting two different models

Model #	Predictors	cNF-Skindex total score		*p*-value
		β	Confidence interval	
1	(Intercept)	27.9	14.8–41.0	**< 0.001**
	> 50 cNF	17.5	2.2–32.8	**0.026**
2	(Intercept)	17.6	−12.1–47.3	0.239
	> 50 cNF	11.4	−5.2–28.0	0.176
	Age	0.4	−0.3–0.8	0.383
	Sex (female)	13.7	−0.9–28.4	0.072
	ISCED level	−3.6	−8.0–0.8	0.105
	Previous cNF-related intervention	11.4	−5.2–28.0	0.130

The first model presents an univariate analysis, with the number of cNF being the only variable

The second model presents the results of a multivariate linear regression analysis, controlling for the number of cNF, age, sex, education (ISCED) level, and the occurrence of a previous cNF-related intervention. p-values < 0.05 in bold

## Discussion

This study aimed to translate the cNF-Skindex into Dutch, and to validate this version to enable use in clinical practice and clinical trials. Following psychometric evaluation, the translated version displayed excellent psychometric properties, and the 18 items and three domains of the original French version were confirmed [8].

The Dutch version of the cNF-Skindex showed high internal consistency, which indicates that the items contained in the subdomains and total score are closely related and measure the same construct. Given the excellent test-retest reliability, the results of this questionnaire will be consistent with a low degree of random measurement errors. The convergent validity analysis showed a moderate correlation with the EQ-5D-5L and EQ-5D-5L VAS. The cNF-Skindex correlated as expected with the different scales of the SF-36, with moderate to strong correlations being observed between the total cNF-Skindex score and the SF-36 scales that center around general health, social functioning, emotional well-being, and pain. The results from the convergent validity analysis indicate that cNF-related QoL is mainly entwined with general health perceptions and social-emotional well-being. No noteworthy correlations could be found between the cNF-Skindex and the SF-36 scales of physical functioning, role limitations due to physical health, and energy/fatigue. This was to be expected, since the cNF-Skindex does not contain any items on problems with energy levels, fatigue, or physical health as included in the SF-36 (e.g., the ability of a patient to walk).

It should be noted that the repartition of the number of cNF was skewed in the present study: more than 70% of the participants reported having ≥ 50 cNF. Although there is no clear relationship between the number of cNF and disease severity, this imbalance could have influenced the results of some of the psychometric analyses. E.g. by mainly including patients with a higher number of cNF, the number of floor effects observed for the cNF-Skindex score might be lower compared to a more balanced study population. The number of cNF categories were based on the French cNF-Skindex study, of which the study sample mainly consisted of patients with 1–10 cNF (42%) [8]. Despite the difference in the distribution of the number of cNF between our study and the French study, the psychometric properties of both versions are comparable. This might indicate that the number of cNF does not significantly impact the psychometric properties of the cNF-Skindex.

The exploratory factor analysis confirmed the 18 items and the three subdomains ‘Functioning’, ‘Emotions’, and ‘Symptoms’ of the original French study [8]. Our results classified item 11 “Feeling depressed about your cNF” under the ‘Functioning’ domain, while it belonged to the domain ‘Emotions’ in the original French investigation. However, in the French validation study, this item also showed a considerable factor loading in the ‘Functioning’ domain (0.38), loading just slightly more on the ‘Emotions’ domain (0.47). In addition, as the domain ‘Functioning’ assesses the disturbances in social life [8], it is likely that feelings of depression affect the social life of the patient, and as such causes this item to load on both factors.

Previous studies showed that a higher number of cNF correlates with increased Skindex and cNF-Skindex scores and impaired cNF-related QoL [7, 8, 19]. Although the mean cNF-Skindex score did increase with the number of cNF, and having > 50 cNF significantly affected the cNF-Skindex score in univariate analysis, we were not able to confirm this finding in multivariate linear regression. This could be explained by the skewed repartition of the number of cNF in the present study. The small sample size of the present study could also be an explanation. To reach a statistical power of 80% for the multivariate linear regression analysis, it would have required a sample size of 91 participants, and we included only 59 participants.

Other factors also did not significantly influence cNF-Skindex scores, including education level, age, sex, and the occurrence of cNF-related interventions. This is unfortunate, considering that the identification of influencing factors could aid medical decision making and clinical trial design for cNF. Factors that influence cNF-related QoL could help predict which patients are likely to be more severely affected by their condition, and which patient could potentially require treatment. It could also aid the identification of modifiable factors and treatment targets: e.g. if a higher number of cNF is associated with impaired cNF-related QoL, reducing the number of cNF could be a meaningful trial endpoint. Given that the present study was not powered to detect such factors, further studies would be valuable to study the influence of relevant patient characteristics on the cNF-Skindex score.

Before the development of the cNF-Skindex, past studies have used various PROs to study cNF-related QoL, including generic PROs like the SF-36 [10], and dermatology-specific PROs such as the Skindex-29 [7]. While generic measures might be necessary to compare QoL across different disease populations, they might not cover distinct symptoms that are relevant to a specific disease. Disease-specific PROs also might be more sensitive to capture (small) changes in QoL over time, especially when patients are treated for disease-specific symptoms [11]. Given the increased recognition of the importance to include PROs as outcome measures in clinical trials [9, 27], the need for a specific PRO for this condition was evident. The cNF-Skindex is the first PRO that specifically measures cNF-related QoL in patients with NF1. It will be a valuable and, as confirmed in the present study, feasible and reliable outcome measure in future trials for NF1-related cNF, providing insight in a specific domain of QoL that is very relevant to both patients and clinicians.

A limitation of this study is the small sample size. The Erasmus MC is an NF1 expertise center that cares for more than 1000 adult patients with NF1, but it still proved difficult to recruit a large number of patients. A considerable number of patients indicated that they would rather not participate, and not all of the patients who expressed interest to participate completed the questionnaires. In addition, there might have been some selection bias, as significantly more female patients participated in this study. We also did not know the extent of the learning difficulties of the participants, as this was self-reported as being present yes or no. The presence of cognitive impairment might complicate the completion of PRO questionnaires for more severely affected patients. As such, we corrected for educational level in multivariate linear regression, although it did not appear to have a significant influence on the cNF-Skindex scores.

## Conclusions

We translated the cNF-Skindex into Dutch and subsequently validated this version in a sample of Dutch patients with NF1 and at least one cNF. The Dutch language version showed high levels of feasibility, reliability, and validity, making it possible to reliably measure the cNF-related QoL of patients with NF1 in the Netherlands. Further trans-cultural validation of the cNF-Skindex will be of utmost importance, since multinational trials are often a necessity in rare conditions like NF1. Given the nearly identical psychometric properties of the French and Dutch version, we are expectant that the cNF-Skindex will show adequate psychometric properties in other trans-cultural validations.

### Electronic supplementary material

Below is the link to the electronic supplementary material.

Supplementary Material 1

## Data Availability

The datasets generated and/or analysed during the current study are not publicly available due to sensitivity of the data and the risk of compromising the individual privacy of the participants. The analysed data is only available from the corresponding author on reasonable request.

## Abbreviations

cNF: Cutaneous neurofibroma
IRB: Institutional review board
KMO: Kaiser–Meyer–Olkin
NF1: Neurofibromatosis type 1
PI: Principal investigator
PRO: Patient-reported outcome
QoL: Quality of life
