# Supplementary material for: Quality of life in individuals with neurofibromatosis type 1 associated cutaneous neurofibromas: validation of the Dutch cNF-Skindex
Source: J Patient Rep Outcomes. 2024 May 29;8:52. doi: 10.1186/s41687-024-00732-w (PMC11136889; doi:10.1186/s41687-024-00732-w)
Supplement: Supplementary file 1 — Supplementary Material 1 [file 41687_2024_732_MOESM1_ESM.pdf]

ANNEX 1 - Correlation between the total and subdomain scores of the Dutch cNF-Skindex with the SF-36 scales

Values in bold: significant correlation at the $p < 0.05$ level.

		cNF-Skindex				SF-36							
		Total score	Functioning subdomain	Emotions subdomain	Symptoms subdomain	Physical functioning	Role limitations due to physical health	Role limitations due to emotional problems	Energy/fatigue	Emotional well-being	Social functioning	Pain	General health
cNF-Skindex	Total score	1.000											
	Functioning subdomain	**0.921**	1.000										
	Emotions subdomain	**0.941**	**0.820**	1.000									
	Symptoms subdomain	**0.835**	**0.665**	**0.699**	1.000								
SF-36	Physical functioning	-0.193	-0.243	-0.050	**-0.275**	1.000							
	Role limitations due to physical health	-0.212	-0.219	-0.105	**-0.278**	**0.542**	1.000						
	Role limitations due to emotional problems	**-0.370**	**-0.335**	**-0.302**	**-0.412**	-0.140	**0.439**	1.000					
	Energy/fatigue	-0.070	-0.113	0.025	-0.111	**0.573**	**0.659**	**0.382**	1.000				
	Emotional well-being	**-0.338**	**-0.404**	**-0.300**	-0.163	0.085	0.248	**0.505**	**0.486**	1.000			
	Social functioning	**-0.426**	**-0.445**	**-0.315**	**-0.428**	**0.492**	**0.618**	**0.622**	**0.574**	**0.539**	1.000		
	Pain	**-0.309**	-0.221	-0.181	**-0.454**	**0.512**	**0.542**	**0.280**	0.385	0.074	**0.508**	1.000	
	General health	**-0.354**	**-0.393**	**-0.261**	**-0.325**	**0.598**	**0.559**	**0.383**	**0.604**	**0.375**	**0.536**	**0.452**	1.000

ANNEX 2 - Results of the exploratory factor analysis with promax rotation

**Item**		**Factor 1**	**Factor 2**	**Factor 3**
1	Your cNF itching	0.162	**0.783**	-0.087
2	Your cNF burning or stinging	0.127	**0.660**	0.135
3	Your cNF hurting	0.306	**0.730**	-0.098
4	Your cNF being irritated	0.154	**0.779**	-0.065
5	The persistence / reoccurrence of your cNF	**0.698**	0.250	-0.077
6	Worry about your cNF	**0.668**	0.403	-0.169
7	The appearance of your cNF	**0.664**	0.209	0.047
8	Frustration about your cNF	**0.960**	-0.137	0.096
9	Embarrassment about your cNF	**0.915**	-0.088	0.099
10	Being annoyed about your cNF	**0.771**	-0.040	0.157
11	Feeling depressed about your cNF	**0.736**	0.036	0.229
12	The effects of your cNF on your interactions with others	0.016	-0.125	**0.985**
13	The effects of your cNF on your desire to be with people	0.349	-0.249	**0.799**
14	Your cNF making it hard to show affection	0.163	0.158	**0.580**
15	The effects of your cNF on your daily activities	0.206	0.260	**0.614**
16	Your cNF making it hard to work or do what you enjoy	-0.033	0.234	**0.761**
17	Your cNF changing how touch, heat, or cold feel on your skin	-0.009	**0.902**	-0.091
18	Your cNF gripping your clothes or when brushing your hair	-0.486	**0.801**	0.422
Eigenvalues		10.7	1.8	1.3
% of variance		59.3%	9.8%	7.3%

Table A1. Results of the exploratory factor analysis: Eigenvalues and % of variance explained of selected factors, and component matrix.

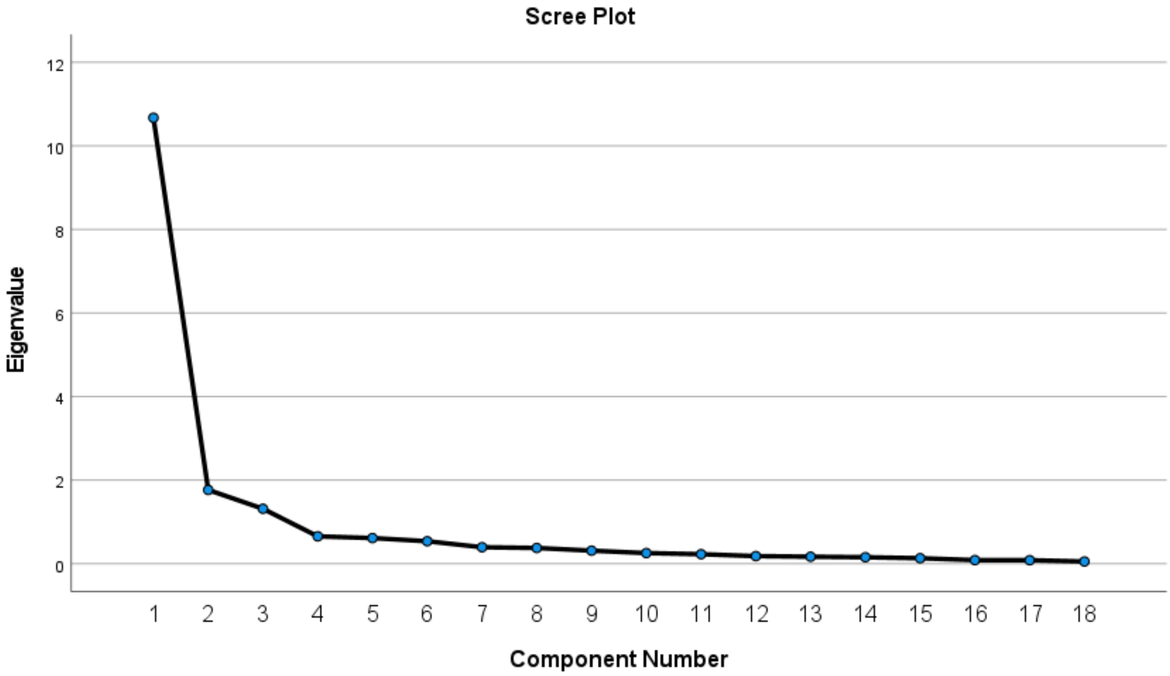

Figure A1. The scree plot belonging to the exploratory factor analysis.

Factor	Eigenvalues
1	10.673
2	1.767
3	1.316
4	.656
5	.615
6	.539
7	.395
8	.378
9	.313
10	.255
11	.229
12	.183
13	.168
14	.155
15	.133
16	.087
17	.085
18	.054

Table A2. Eigenvalues of all factors of the exploratory factor analysis.
